# Preexisting respiratory diseases and clinical outcomes in COVID-19: a multihospital cohort study on predominantly African American population

**DOI:** 10.1186/s12931-021-01647-6

**Published:** 2021-02-05

**Authors:** Prateek Lohia, Kalyan Sreeram, Paul Nguyen, Anita Choudhary, Suman Khicher, Hossein Yarandi, Shweta Kapur, M. Safwan Badr

**Affiliations:** 1 Department of Internal Medicine, Wayne State University, 4201 St Antoine, Detroit, MI UHC 5C USA; 2 Wayne State University, Detroit, MI USA

**Keywords:** COVID-19, Mechanical ventilation, Intensive care, Smoking, tobacco, Mortality

## Abstract

**Background:**

Comorbidities play a key role in severe disease outcomes in COVID-19 patients. However, the literature on preexisting respiratory diseases and COVID-19, accounting for other possible confounders, is limited. The primary objective of this study was to determine the association between preexisting respiratory diseases and severe disease outcomes among COVID-19 patients. Secondary aim was to investigate any correlation between smoking and clinical outcomes in COVID-19 patients.

**Methods:**

This is a multihospital retrospective cohort study on 1871 adult patients between March 10, 2020, and June 30, 2020, with laboratory confirmed COVID-19 diagnosis. The main outcomes of the study were severe disease outcomes i.e. mortality, need for mechanical ventilation, and intensive care unit (ICU) admission. During statistical analysis, possible confounders such as age, sex, race, BMI, and comorbidities including, hypertension, coronary artery disease, congestive heart failure, diabetes, any history of cancer and prior liver disease, chronic kidney disease, end-stage renal disease on dialysis, hyperlipidemia and history of prior stroke, were accounted for.

**Results:**

A total of 1871 patients (mean (SD) age, 64.11 (16) years; 965(51.6%) males; 1494 (79.9%) African Americans; 809 (43.2%) with ≥ 3 comorbidities) were included in the study. During their stay at the hospital, 613 patients (32.8%) died, 489 (26.1%) needed mechanical ventilation, and 592 (31.6%) required ICU admission. In fully adjusted models, patients with preexisting respiratory diseases had significantly higher mortality (adjusted Odds ratio (aOR), 1.36; 95% CI, 1.08–1.72; *p* = 0.01), higher rate of ICU admission (aOR, 1.34; 95% CI, 1.07–1.68; *p* = 0.009) and increased need for mechanical ventilation (aOR, 1.36; 95% CI, 1.07–1.72; *p* = 0.01). Additionally, patients with a history of smoking had significantly higher need for ICU admission (aOR, 1.25; 95% CI, 1.01–1.55; *p* = 0.03) in fully adjusted models.

**Conclusion:**

Preexisting respiratory diseases are an important predictor for mortality and severe disease outcomes, in COVID-19 patients. These results can help facilitate efficient resource allocation for critical care services.

## Introduction

Coronavirus disease-2019 (COVID-19) has infected close to 55.6 million people worldwide and resulted in more than 1.34 million deaths as of late-November 2020. In the United States (US) alone, more than 11.6 million people have been infected and 250,000 people have died. Early reports from the US suggest that patients with preexisting comorbid diseases including chronic lung diseases are at a higher risk of severe COVID-19 disease [1–3]. Similar studies from China [4–7] and Italy [8] have noted that patients with preexisting respiratory diseases have higher mortality. According to the global burden of disease, Chronic Obstructive Pulmonary Disease (COPD) is the third leading cause of death worldwide [9] and chronic lower respiratory diseases have been identified as the fourth leading cause of death in the US accounting for 5.7% of total deaths [10]. Obstructive sleep apnea (OSA) is another common preexisting respiratory condition affecting close to 1 billion people worldwide, with high prevalence in the US [11]. However limited information is available describing mortality and the need for mechanical ventilation in patients with preexisting respiratory diseases and COVID-19.

A study by the Chinese Center for Disease Control and Prevention had reported an average case-fatality rate of around 2.3%, however, significantly higher mortality was noted in critically ill patients in intensive care [4]. Other studies have reported that 2–3% of patients infected with COVID-19 require mechanical ventilation [12–14] and reported a case fatality rate of 1.2% in the US [15].

Literature is abundant on the negative impact of smoking on lung health and its association with a plethora of respiratory conditions. Smoking is also detrimental to the immune system [16] and its response to various infections. Studies have delineated the implications of increased risk of infection among smokers [17]. Recently an association of smoking with negative progression and adverse outcomes in COVID-19 patients has been reported [18].

The increased number of COVID-19 patients presenting with critical illness has resulted in limited availability of intensive care beds and strained hospital resources [19]. It is important to identify patients who are at risk for critical illness, need intensive care and mechanical ventilation to optimize the use of critical care resources, especially in inner-city and predominantly underserved areas. It can aid in efficient resource allocation, planning for critical care surge, and appropriate deployment of health care workers.

The main objective of this study is to determine the correlation between preexisting respiratory diseases and severe disease outcomes i.e. mortality, need for mechanical ventilation, and intensive care unit (ICU) admission among COVID-19 patients. Our study also explores if the history of smoking in COVID-19 patients is associated with the severe disease outcomes mentioned above.

## Methods

### Study design

We conducted a retrospective cohort study on 1871 adult patients with confirmed COVID-19 diagnosis. This study was deemed exempt by the Detroit Medical Center (DMC) and Wayne State University institutional review board. (IRB application #20-07-2528). No external funding was received for conducting the study.

### Study site and patient population

Adult patients (≥ 18 years of age) with a confirmed COVID-19 diagnosis (either via nasopharyngeal or oropharyngeal swab) were included. Testing for COVID-19 was done at the DMC, one of the largest academic medical centers and healthcare providers in Southeast Michigan. DMC comprises four distinct hospitals in Michigan and data from all four hospitals have been included in this study. These hospitals primarily serve the Detroit metropolitan area catering to an underserved population majority of which is African American.

### Data collection

A list of patients was collected in collaboration with institutional information technology services. Patients who visited DMC between March 10, 2020, and June 30, 2020, with a laboratory confirmed COVID-19 PCR diagnosis were included. Patients under the age of 18, any readmission during the time frame, ambulatory surgery patients, and pregnant patients were excluded from the study. Patients who were transferred to an outside facility for extracorporeal membrane oxygenation (ECMO) therapy were also excluded.

To determine preexisting respiratory diseases and smoking status, along with other variables, we manually searched through clinical notes, emergency department (ED) notes, and prior history tab in the electronic medical records (EMR). Preexisting respiratory diseases included in the study were COPD, asthma, pulmonary hypertension, OSA, pulmonary embolism, sarcoidosis, lung cancer, prior tuberculosis, and interstitial lung disease. Data points were manually collected and coded for each patient. Data regarding radiographic imaging during hospitalization, initial chest X-ray and chest computerized tomography (CT) scan were also collected for all the patients, whenever available. The severity of the preexisting respiratory diseases was also noted, if the information was available. Disease severity for each condition was determined as follows: (a) COPD severity was based on the GOLD grade using the pulmonary function tests (PFTs), (b) OSA severity was classified based on the apnea–hypopnea index (AHI) from the sleep studies, (c) asthma severity was determined based upon symptoms, nocturnal awakening and PFT’s (d) pulmonary hypertension, based on mean pulmonary arterial pressure on right heart catheterization, and (e) sarcoidosis, based on the baseline chest X-ray findings. Positive smoking status was established based on the documented smoking history on the review of EMR. Quantification of the amount of smoking and categorization of smokers into current and former smokers could not be done due to the lack of consistent documentation in EMR. Also, the nature and clinical course of the patient’s hospitalization and their disposition from the ED visit were noted.

### Outcomes

The main outcomes for this study were mortality, need for mechanical ventilation, and ICU admission. Together, they have been referred to as severe disease outcomes in COVID-19. All of the patients included in the study had a documented acute care endpoint (mortality/discharged status) at the time of data collection. Additionally, the number and type of prior comorbidities, BMI, disposition upon ED visit (discharge home, inpatient admission, and direct ICU admission) were collected. Data regarding whether or not the patient received corticosteroid treatment during the course of their hospitalization were also obtained. Charts were screened to determine if the patient required up-gradation of care to the ICU from inpatient floors. Demographic data collected included age, sex, and race.

### Statistical analysis

Categorical variables have been described as frequency and percentages, and continuous variables have been described as mean and standard deviation. A crude relative association measure (Odds ratio, OR) was calculated for each correlation using the Pearson chi-square and Fisher test. An adjusted odds ratio was calculated using binary logistic regression. In the fully adjusted models, adjustments were made for age, sex, race, BMI, and prior comorbidities including, hypertension, coronary artery disease (CAD), congestive heart failure (CHF), diabetes, any history of cancer and prior liver disease, chronic kidney disease (CKD), end-stage renal disease (ESRD) on dialysis, hyperlipidemia and history of prior stroke. Age and BMI were taken as continuous variables while the remaining were categorical variables. A p-value of less than 0.05 was determined to be significant. Stepwise regression using forward selection (Wald) method was also performed to obtain an optimal model and further validate the findings. Subgroup analyses were done based on the type of preexisting respiratory disease. Analysis based on the severity of preexisting respiratory disease could not be conducted due to the non-availability of this data for a large number of patients. Statistical analyses were completed using IBM SPSS Statistics software (version 26).

## Results

### Baseline characteristics

There were 2001 adult patient records with positive COVID-19 test at the 4 DMC hospitals with a nasopharyngeal/oropharyngeal PCR swab between March 10, 2020, and June 30, 2020. A total of 130 patient records were excluded based on the exclusion criteria, and 1871 patients were included in the study. In the cohort analysis, there was an almost equal distribution of males (n = 965, 51.6%) and females (n = 906, 48.4%). The mean age of patients was 64.11 years (Standard deviation SD 16). More than half the patients (n = 997, 53.3%) were 65 years or older, with African Americans being the predominant race (n = 1494, 79.9%). About 43% of the patients had three or more comorbid diseases (n = 809). The mean BMI of the patient cohort was 31.14 kg/m^2^ (SD 8.82) and 47% (n = 897) patients were in the obese category, 23 patients were missing BMI information in the chart. About 30.7% of all the patients (n = 575) had a documented preexisting respiratory disease as part of their medical history. Additionally, 37.6% (n = 704) of patients had a history of smoking identified as a part of their social history. The baseline characteristics of the population included are detailed in Table 1.

**Table 1:** Baseline characteristics of patients

Characteristics	Cohort (n = 1871)
**Age**, n (%)	
Mean (SD)	64.11 (16)
< 65	874 (46.7)
≥ 65	997 (53.3)
**Sex**, n (%)	
Male	965 (51.6)
Female	906 (48.4)
**Race/ethnicity**, n (%)	
African American	1494 (79.9)
White	340 (18.2)
Asian	21 (1.1)
Middle Eastern	14 (0.7)
Latino/Hispanic	2 (0.1)
**BMI**, mean (SD)	31.14 (8.82)
< 18.5 (underweight)	46 (2.5)
18.5–24.9 (normal)	411 (22)
25–29.9 (overweight)	512 (27.4)
≥ 30 (obese)	897 (47)
**Preexisting respiratory disease**, n (%)	575 (30.7)
COPD	317 (16.9)
Asthma	134 (7.2)
Obstructive sleep apnea	63 (3.4)
Pulmonary embolism	27 (1.4)
Pulmonary hypertension	10 (0.5)
Sarcoidosis	8 (0.4)
Lung cancer	9 (0.5)
Prior TB/ILD	5 (0.3)
**Number of comorbidities**, n (%)	
0	257 (13.7)
1	362 (19.3)
2	443 (23.7)
≥ 3	809 (43.2)
**Current or former smoker**, n (%)	704 (37.6)
**Individual preexisting respiratory disease severity**	
** COPD**, n (%)	317
GOLD grade I	40 (12.6)
GOLD grade II	18 (5.7)
GOLD grade III	16 (5)
GOLD grade IV	5 (1.6)
Cannot be determined	238 (75.1)
**Asthma**, n (%)	134
Intermittent	9 (6.7)
Mild	13 (9.7)
Moderate	5 (3.7)
Severe	2 (1.5)
Cannot be determined	105 (78.4)
**Obstructive sleep apnea**, n (%)	63
Mild (5 ≤ AHI < 15)	7 (11.1)
Moderate (15 ≤ AHI < 30)	6 (9.5)
Severe (AHI ≥ 30)	18 (28.6)
Cannot be determined	32 (50.8)
**Pulmonary Hypertension** (based on mPAP), n (%)	10
Mid	4 (40)
Moderate	1 (10)
Severe	2 (20)
Cannot be determined	3 (30)
**Sarcoidosis**, n (%)	8
Stage 0	3 (37.5)
Stage 1	2 (25)
Cannot be determined	3 (37.5)

*SD* Standard deviation, *AHI* Apnea–hypopnea index, *mPAP* Mean pulmonary arterial pressure

*mPAP* Mean pulmonary arterial pressure

### Clinical course

The total mortality in the cohort was 32.8% (n = 613). About 17.5% (n = 327) patients were admitted directly to ICU from the ED. An additional 265 were later transferred to ICU from the inpatient service. Approximately one in every three patients (31.6%) who presented to ED ended up requiring ICU services. Around 8.8% of the total patients were sent home from ED (n = 165), while 73.7% (n = 1379) were admitted to the inpatient service. During the course of hospitalization, 26.1% of the patients (n = 489) required mechanical ventilation. Unilateral/bilateral infiltrates on chest X-ray at admission was the most common radiographical finding. Further details on the clinical course of the patients and radiographical findings are summarized in Table 2.

**Table 2:** Clinical course of patients (cohort n = 1871)

Mortality	613 (32.8)
Mechanical ventilation	489 (26.1)
ICU admission	592 (31.6)
Admission disposition	
ER Visit Only (Discharged from ER)	165 (8.8)
Inpatient Admission	1379 (73.7)
Direct ER to ICU admission	327 (17.5)
Chest x-ray at admission	1821
Infiltrates (unilateral/bilateral)	1242 (68.2)
Atelectasis	208 (11.4)
Pleural effusion	31 (1.7)
Pulmonary vascular congestion/edema	106 (5.8)
Normal	234 (12.9)
CT scan findings during admission	93
Consolidation	15 (16.1)
Ground glass opacities	59 (63.4)
Pulmonary infiltrates (unilateral/bilateral)	11 (11.8)
Interstitial abnormalities (reticular, fibrous stripes, interlobular septal thickening)	7 (7.5)
Normal	1 (1.1)
Corticosteroids during admission	571
Preexisting respiratory disease	230 (40.3)
No preexisting respiratory disease	341 (59.7)

### Preexisting respiratory disease and severe disease outcomes

Patients with preexisting respiratory diseases had significantly higher mortality, higher need for ICU admission, and a greater need for mechanical ventilation, compared to the patients without preexisting respiratory diseases. In unadjusted analysis, patients with preexisting respiratory disease were associated with higher mortality, compared to those without any preexisting respiratory disease (OR = 1.29; 95% CI, 1.05–1.58; *p* = 0.02). Having a preexisting respiratory disease was also associated with a higher rate of ICU admission (OR, 1.33; 95% CI, 1.08–1.64; *p* = 0.007) as well as increased need for mechanical ventilation (OR, 1.40; 95% CI, 1.13–1.74; *p* = 0.002).

Even after adjusting for age, sex, race, BMI, and prior comorbidities including, hypertension, CAD, CHF, diabetes, any history of cancer and prior liver disease, CKD, ESRD on dialysis, hyperlipidemia, and history of prior stroke, patients with preexisting respiratory diseases had higher mortality (adjusted(a)OR = 1.36; 95% CI, 1.08–1.72; *p* = 0.01), increased need for ICU admission (aOR = 1.34; 95% CI, 1.07–1.68; *p* = 0.009), and higher rates of requiring mechanical ventilation (aOR = 1.36; 95% CI, 1.07–1.72; *p* = 0.01). Further details on the results of unadjusted models and fully adjusted models for the association between preexisting respiratory disease and the three severe disease outcomes are outlined in Table 3. The results for stepwise regression models exploring the association between preexisting respiratory disease and the clinical outcomes have been summarized in Table 4.

**Table 3:** Association between preexisting respiratory disease/smoking and severe disease outcomes- Mortality, Mechanical ventilation and ICU admission unadjusted and adjusted for age, sex, race, BMI and comorbidities

Characteristic	Mortality		ICU Admission		Mechanical ventilation	
	OR (95% CI)	*p*-value	OR (95% CI)	*p*-value	OR (95% CI)	*p*-value
Preexisting respiratory disease						
Unadjusted	1.29 (1.05–1.58)	0.02	1.33 (1.08–1.64)	0.007	1.40 (1.13–1.74)	0.002
Fully adjusted*	1.36 (1.08–1.72)	0.01	1.34 (1.07–1.68)	0.009	1.36 (1.07–1.72)	0.01
Smoking						
Unadjusted	1.26 (1.03–1.53)	0.02	1.33 (1.09–1.62)	0.005	1.23 (0.99–1.52)	0.05
Fully adjusted*	1.14 (0.91–1.42)	0.25	1.25 (1.01–1.55)	0.03	1.15 (0.92–1.44)	0.21

*Fully adjusted for age, sex, race, BMI and comorbidities which include hypertension, coronary artery disease, diabetes, chronic kidney disease, ESRD on dialysis, congestive heart failure, any cancer, any liver disease, hyperlipidemia and history of previous stroke

*OR* odds ratio, *CI* Confidence Interval

**Table 4:** Association between preexisting respiratory disease/smoking and severe disease outcomes- Mortality, Mechanical ventilation and ICU admission (using stepwise regression, forward selection Wald approach)

Mortality		ICU Admission		Mechanical ventilation	
OR (95% CI)	*p*-value	OR (95% CI)	*p*-value	OR (95% CI)	*p*-value
Preexisting respiratory diseases					
1.38 (1.10–1.74)*	0.005	1.34 (1.08–1.66)**	0.009	1.34 (1.07–1.69)***	0.01
Smoking					
NS		1.28(1.04–1.57)^	0.02	NS	

*Variables in the optimal model- age, sex, BMI, diabetes, chronic kidney disease and preexisting respiratory diseases

**Variables in the optimal model- age, sex, BMI, diabetes, chronic kidney disease and preexisting respiratory diseases

***Variables in the optimal model- age, sex, BMI, diabetes, hypertension, and preexisting respiratory diseases

^Variables in the optimal model- age, sex, BMI, diabetes, chronic kidney disease and smoking

*OR* odds ratio, *CI* confidence interval, *NS* nonsignificant

### Type of preexisting respiratory disease and severe disease outcomes

Among patients with preexisting respiratory diseases, the most prevalent condition was COPD, present in more than half of the patients (n = 317). In the unadjusted models, COPD (OR, 1.47; 95% CI, 1.14–1.88; *p* = 0.002), Asthma (OR, 0.57; 95% CI, 0.38–0.87; *p* = 0.008) and OSA (OR, 2.04; 95% CI, 1.23–3.37; *p* = 0.005) demonstrated significant association with mortality. The need for mechanical ventilation was also significantly higher for patients with COPD (OR, 1.35; 95% CI, 1.04–1.76; *p* = 0.02) and OSA (OR, 2.85; 95% CI, 1.72–4.73; *p* < 0.001). In fully adjusted models, however, only the association of OSA with the three severe disease outcomes was found to be statistically significant, mortality (aOR, 2.59; 95% CI, 1.46–4.58; p = 0.001), ICU admission (aOR, 1.95; 95% CI, 1.14–3.32; *p* = 0.01) and need for mechanical ventilation (aOR, 2.20; 95% CI, 1.28–3.78; *p* = 0.004). Table 5 summarizes the association of different preexisting respiratory diseases with the severe disease outcomes in the unadjusted as well as the fully adjusted models.

**Table 5:** Association between individual preexisting respiratory disease and severe disease outcomes- Mortality, Mechanical ventilation and ICU admission

Characteristic	Number of events n (%)	Unadjusted		Fully adjusted*	
		OR (95% CI)	*p*-value	OR (95% CI)	*p*-value
Mortality					
COPD	127 (40.1)	1.47 (1.14–1.88)	0.002	1.20 (0.91–1.58)	0.2
Asthma	30 (22.4)	0.57 (0.38–0.87)	0.008	0.98 (0.61–1.58)	0.94
Obstructive sleep apnea	31 (49.2)	2.04 (1.23–3.37)	0.005	2.59 (1.46–4.58)	0.001
Pulmonary embolism	13 (48.1)	1.92 (0.90–4.12)	0.09	1.86 (0.82–4.23)	0.14
Pulmonary hypertension	4 (40)	1.37 (0.38–4.87)	0.62	1.09 (0.28–4.23)	0.9
Sarcoidosis	1 (12.5)	0.29 (0.04–2.38)	0.28	0.39 (0.04–3.46)	0.4
Lung cancer	4 (44.4)	1.65 (0.44–6.15)	0.45	1.24 (0.30–5.10)	0.76
ICU admission					
COPD	114 (36)	1.26 (0.98–1.63)	0.07	1.20 (0.92–1.58)	0.18
Asthma	41 (30.6)	0.95 (0.65–1.39)	0.79	1.16 (0.77–1.74)	0.47
Obstructive sleep apnea	32 (50.8)	2.3 (1.39–3.80)	0.001	1.95 (1.14–3.32)	0.01
Pulmonary embolism	13 (48.1)	2.30 (0.95–4.34)	0.06	2.03 (0.93–4.44)	0.08
Pulmonary hypertension	4 (40)	1.44 (0.41–5.13)	0.57	1.25 (0.34–4.62)	0.74
Sarcoidosis	1 (12.5)	0.31 (0.04–2.50)	0.49	0.39 (0.05–3.21)	0.38
Lung cancer	2 (22.2)	0.62 (0.13–2.97)	0.54	0.63 (0.12–3.28)	0.58
Mechanical ventilation					
COPD	99 (31.2)	1.35 (1.04–1.76)	0.02	1.28 (0.96–1.69)	0.09
Asthma	33 (24.6)	0.92 (0.61–1.38)	0.68	1.08 (0.69–1.67)	0.74
Obstructive sleep apnea	31 (49.2)	2.85 (1.72–4.73)	< 0.001	2.20 (1.28–3.78)	0.004
Pulmonary embolism	9 (33.3)	1.42 (0.63–3.18)	0.39	1.45(0.63–3.34)	0.39
Pulmonary hypertension	3 (30)	1.21 (0.31–4.70)	0.72	0.96 (0.23–3.92)	0.95
Sarcoidosis	1 (12.5)	0.40 (0.0–3.28)	0.69	0.52 (0.06–4.30)	0.54
Lung cancer	1 (11.1)	0.35 (0.04–2.82)	0.46	0.35 (0.04–3.00)	0.34

*Fully adjusted for age, sex, race, BMI and comorbidities which include hypertension, coronary artery disease, diabetes, chronic kidney disease, ESRD on dialysis, congestive heart failure, any cancer, any liver disease, hyperlipidemia and history of previous stroke

*OR* odds ratio, *CI* Confidence Interval

### Smoking and severe disease outcomes

Smoking was associated with higher mortality (OR, 1.26; 95% CI, 1.03–1.53; *p* = 0.02) and increased need for ICU admission (OR, 1.33; 95% CI, 1.09–1.62; *p* = 0.005). The association between smoking and the need for mechanical ventilation was not statistically significant (OR, 1.23; 95% CI, 0.99–1.52; *p* = 0.05). After adjusting for age, sex, race, BMI, and comorbidities, a significant association was only noted between smoking and ICU requirement (aOR, 1.25; 95% CI, 1.01–1.55; *p* = 0.03). Table 3 outlines the association of smoking with severe disease outcomes.

## Discussion

This retrospective cohort study provides novel findings indicating the role of preexisting respiratory diseases as an important predictor of severe disease outcomes in patients hospitalized with COVID-19. The study demonstrated a significant association between the presence of preexisting respiratory diseases and mortality, ICU admission, and need for mechanical ventilation. Even when adjusted for possible confounders such as age, sex, race, BMI and ten prevalent comorbidities, patients with preexisting respiratory disease had significantly higher mortality, greater need for ICU admission, and increased need for mechanical ventilation. Hence, the study demonstrates that preexisting respiratory diseases are an important predictor for severe disease outcomes in COVID 19 patients.

Hypertension, coronary artery disease, and diabetes are the most common reported comorbidities among COVID-19 patients [6, 20–24], and they have been found to be associated with severe disease outcomes. Studies from China [5, 12, 13, 25–27] and Italy [28] have reported that patients with chronic lung diseases have worse clinical outcomes, however, they evaluated a much smaller cohort. Obesity also has been reported by some, to be a risk factor for mortality in COVID-19 [20, 29, 30]. To date, the literature on the role of preexisting respiratory conditions in the clinical course of COVID-19 positive patients has been limited, and our study highlights that the presence of preexisting respiratory diseases has a significant impact on clinical outcomes.

To our knowledge, this is the first study that has looked at the association of all the prominent respiratory diseases with severe disease outcomes in COVID-19 patients. Patients with OSA had significantly higher mortality, a higher need for mechanical ventilation, and a greater need for ICU admission in our study. A recent study by Cade et al. [31] also noted a significant crude association between sleep apnea and mortality. However, in their study, the associations were somewhat attenuated after adjusting for BMI and other comorbidities. Another study by Maas et al. [32] reported that OSA was associated with an increased risk of hospitalization and approximately double the risk of developing respiratory failure. The patients with OSA in our study were also more than twice as likely to require mechanical ventilation, compared to the patients without OSA. Prior diagnosis of OSA in COVID-19 patients has also been reported to be associated with increased risk of death at day 7 [33]. A review by Miller et al. [34] provides a plausible explanation linking OSA and COVID-19. It hypothesizes that periods of hypercapnia and hypoxemia, surges of sympathetic activation, and increased inflammatory markers in OSA, may contribute to worse outcomes in COVID-19 patients. Further research is warranted to better understand the mechanism by which OSA might be contributing to worse clinical outcomes in COVID-19 patients.

In our study, patients with COPD also had increased mortality and a higher need for mechanical ventilation. However, upon adjusting for age, sex, race, BMI, and comorbidities, associations were attenuated and failed to reach the level of traditional significance. In the study by Grasselli et al. [28] COPD was noted to be significantly associated with mortality in multivariable analysis, however, this study did not adjust for BMI which could be a possible confounder and was accounted for in our study. Also, in their cohort of 3988 ICU patients, only 0.02% of the patients had COPD, thereby one can surmise that COPD does not have a significant association with the higher need for ICU admission, as seen in our study. We were unable to demonstrate any statistically significant correlation between other respiratory conditions, apart from COPD and OSA, and the severity outcomes explored by this study. This may be, in part, due to the far smaller sample sizes for these other respiratory conditions in our cohort.

This study also demonstrated a crude association between smoking and severe disease outcomes, particularly with mortality and the need for intensive care services. Similar studies looking at the association of smoking have also demonstrated worse clinical outcomes in patients with COVID-19[5, 13], increased rate of hospitalizations [35] and increased incidence of COVID-19 among young adults [36]. Recent literature shows an association of smoking and expression of angiotensin converting enzyme-2 (ACE-2) in small airway epithelia [37, 38], which has been identified as the cell entry receptor for the SARS-CoV 2 virus [39–41]. A recent meta-analysis done by Karanasos et. al. [42] showed smoking modestly increased disease severity in COVID-19 patients, similar to what has been reported by our study. However, vast majority of the studies included in this meta-analysis did not adjust for confounders. In our study, when we controlled for age, sex, race, comorbidities, and BMI, we still noted a significant association between smoking and the need for ICU admission.

Our study has several limitations that must be acknowledged. The data collected relied on clinical notes to gather the history of preexisting respiratory disease and smoking. It is subject to both selection and information bias. Although we had a large database of 2000 + patients, the number of patients with certain preexisting respiratory diseases such as OSA, Pulmonary Hypertension, Sarcoidosis, lung cancer was relatively small. Also, this is a retrospective study on the data from 4 hospitals in a single geographic location, predominantly serving the underserved population with a majority of patients being African American, having multiple comorbidities. This may limit the generalization of these results. We could not explore further if the severity of respiratory disease had any impact on COVID-19 disease progression or clinical outcomes since the data used to determine the severity of preexisting respiratory diseases were not available for a large number of patients in this cohort. Another limitation of our study is the lack of detailed smoking history, including the duration and amount of smoking. Due to the lack of detailed information in the EMR, we could not differentiate between current and former smokers. Therefore, any history of past or current smoking was counted as the smoking status to be positive. Despite these limitations, the findings of this study can help to fill some of the vital voids that currently exist in the understanding of COVID-19.

## Conclusion

Preexisting respiratory diseases are an important comorbid condition associated with worse clinical outcomes, higher mortality, greater need for ICU admission, and increased need for mechanical ventilation, in COVID-19 patients. These results can be useful in planning treatment and allocation of critical care resources, especially during surges, in regions where such resources are limited.

## Data Availability

The deidentified data that support the findings of this study can be available from the corresponding author upon reasonable request and appropriate permission from the institutional IRB.

## Abbreviations

COVID-19: Coronavirus disease
US: United States
COPD: Chronic obstructive pulmonary disease
OSA: Obstructive Sleep Apnea
ICU: Intensive care unit
DMC: Detroit Medical Center
ED: Emergency department
ECMO: Extracorporeal membrane oxygenation
EMR: Electronic medical records
PFT: Pulmonary function test
CAD: Coronary artery disease
CHF: Congestive heart failure
CKD: Chronic kidney disease
ESRD: End stage renal disease
OR: Odds ratio
CI: Confidence interval
SD: Standard Deviation
AHI: Apnea–hypopnea index
mPAP: Mean pulmonary arterial pressure
